# Post-mortem evidence of microplastic bioaccumulation in human organs: insights from advanced imaging and spectroscopic analysis

**DOI:** 10.1007/s00204-025-04092-2

**Published:** 2025-06-25

**Authors:** Eliasz Dzierżyński, Ewelina Blicharz-Grabias, Iwona Komaniecka, Rafal Panek, Alicja Forma, Piotr J. Gawlik, Damian Puźniak, Wojciech Flieger, Adam Choma, Katarzyna Suśniak, Grzegorz Teresiński, Jacek Baj, Krzysztof Kupisz, Jolanta Flieger

**Affiliations:** 1 Department of Plastic Surgery, Center of Oncology of the Lublin Region St. Jana z Dukli, Jaczewskiego 7, 20-090 Lublin, Poland; 2 Department of Analytical Chemistry, Medical University of Lublin, Chodźki 4a (Collegium Pharmaceuticum), 20-093 Lublin, Poland; 3 Department of Genetics and Microbiology, Institute of Biological Sciences, Maria Curie-Sklodowska University, Akademicka 19, 20-033 Lublin, Poland; 4 Department of Geotechnics, Civil Engineering and Architecture Faculty, Lublin University of Technology, Nadbystrzycka 40, 20-618 Lublin, Poland; 5 Department of Forensic Medicine, Medical University of Lublin, Jaczewskiego 8B, 20-090 Lublin, Poland; 6 Institute of Health Sciences, John Paul II Catholic University of Lublin, Konstantynów 1 H, 20-708 Lublin, Poland; 7 Doctoral School, Medical University of Lublin, Aleje Racławickie 1, 20-059 Lublin, Poland; 8 Department of Pharmaceutical Microbiology, Medical University of Lublin, Chodźki 1 St., 20-093 Lublin, Poland; 9 Department of Correct, Clinical and Imaging Anatomy, Medical University of Lublin, Jaczewskiego 4, 20-090 Lublin, Poland; 10 Department of Otorhinolaryngology, Center of Oncology of the Lublin Region St. Jana z Dukli, Jaczewskiego 7, 20-090 Lublin, Poland

**Keywords:** Microplastics, Nanoplastics, Bioaccumulation, Human tissues, Inorganic particles, Synthetic polymers, Advanced spectroscopic, Polymer identification

## Abstract

Humans are chronically exposed to airborne particulate matter and environmental microplastics through food, water, and consumer products. These anthropogenic pollutants may accumulate in human tissues, but their distribution and chemical identity remain poorly understood. In this study, we analyzed samples of human brain, liver, thyroid, kidney, heart, skeletal muscle, and lung tissue collected post-mortem to assess the presence and composition of micro- and nanoplastics (MNPs). Tissue samples were digested using hydrogen peroxide (30% H₂O₂) and processed via alumina filtration. The retained residues and filtrates were characterized using optical microscopy, scanning electron microscopy with energy-dispersive X-ray spectroscopy (SEM–EDS), dynamic light scattering (DLS), matrix-assisted laser desorption/ionization time-of-flight mass spectrometry (MALDI-TOF MS), and optical photothermal infrared (O-PTIR) microscopy. Our analysis revealed a wide range of inorganic particles (primarily aluminosilicates and carbonates) and synthetic polymers, including polyethylene terephthalate (PET), polystyrene (PS), polyacrylonitrile (PAN), and cellulose derivatives. Notably, PS, PET, and PAN nanoparticles (<0.02 µm) were detected in the filtrates, indicating their potential to cross biological barriers and accumulate at the nanoscale. The thyroid, kidney, and brain tissues showed the highest levels of microplastic contamination, with up to 40.4 MP/g (wet weight) detected. These findings confirm the heterogeneous organ-specific accumulation of environmental polymers and highlight the potential of human autopsy tissues as biomonitors for environmental plastic exposure. The application of advanced spectroscopic techniques enables precise identification of polymeric contaminants and supports further research on their environmental origins and pathways of human exposure.

## Introduction

Plastics are among the most widely manufactured materials globally, with production exceeding 300 million tons in 2019 (Kwon 2023). Despite their versatility and irreplaceability in modern life, only around 20% of plastics are recycled, while the remainder accumulates in the environment (Paul et al. 2020; Rangel-Buitrago et al. 2022). As plastics degrade, they fragment into smaller particles—microplastics (<5 mm) and nanoplastics (<1 µm)—which can be dispersed through air, water, and food, and ultimately accumulate in organisms including humans (Arthur et al. 2008; Hartmann et al. 2019; Barceló et al. 2023). 

The COVID-19 pandemic further exacerbated plastic pollution due to the widespread use of single-use synthetic products such as face masks (Kannan and Vimalkumar 2021; Peng et al. 2021). Plastics, composed of carbon-based polymers, frequently contain hazardous additives such as bisphenols, phthalates, and PFAS, which are known to exhibit carcinogenic, neurotoxic, and endocrine-disrupting effects (Landrigan et al. 2023).

In recent years, microplastic exposure in humans has been confirmed by biomonitoring studies that identified synthetic particles in blood, urine, breast milk, placenta, colon, lungs, and other tissues (Ragusa et al. 2022; Leslie et al. 2022; Zuri et al. 2023). Most commonly, microplastics have been analyzed in fecal samples, indicating the potential for ingestion and partial excretion (Schwabl et al. 2019). However, these particles are also capable of translocating across biological barriers, evading immune clearance, and persisting in internal tissues due to their resistance to enzymatic degradation (Paul et al. 2020; Wu et al. 2022).

Microplastics may induce oxidative stress, inflammation, DNA damage, and disruption of barrier integrity, potentially contributing to various chronic diseases (Rahman et al. 2021; Barceló et al. 2023). Despite mounting concerns, most toxicological evidence comes from animal models or in vitro experiments, often under unrealistic exposure scenarios (Deng et al. 2017; Liu et al. 2023). Moreover, microplastics may act as vectors for harmful substances—a phenomenon known as the “Trojan horse” effect (Menéndez-Pedriza et al. 2022).

One of the key challenges in human microplastic research is the lack of standardized methods for detection and quantification, leading to substantial discrepancies between studies (Yan et al. 2022; Jenner et al. 2022). Reliable analysis requires a combination of techniques tailored to particle size, composition, and morphology, such as μFTIR, Raman spectroscopy, SEM/EDS, MALDI-TOF MS, and thermogravimetric methods (Xu et al. 2019; Wu et al. 2020).

In this study, we aimed to investigate the presence and characteristics of environmental microparticles, particularly synthetic polymers, in various human post-mortem tissues, including the brain, liver, thyroid, kidney, heart, lungs, and skeletal muscles. A multimodal analytical approach was applied, combining optical microscopy, SEM/EDS, MALDI-TOF mass spectrometry, and optical photothermal infrared (O-PTIR) microscopy—a cutting-edge technique not previously applied to human tissue samples. Our findings contribute to the understanding of tissue-specific bioaccumulation of microplastics and the analytical challenges in monitoring their distribution in the human body.

## Materials and methods

### Tissue collection

Post-mortem human tissue samples were collected 24 h after death at the Department of Forensic Medicine, Medical University of Lublin, Poland. The subject was a 24-year-old male who died by suicide (hanging), with no prior hospitalization. Autopsy was performed by a certified forensic pathologist. Tissue samples were obtained from the white matter of the brain, thyroid, kidney, liver (sixth intercostal space), pectoralis major muscle, heart (left ventricle), and lung. The study was approved by the Local Bioethics Committee of the Medical University of Lublin (approval no. KE-0254/152/2021, dated 24 June 2021) and conducted in accordance with the Declaration of Helsinki. Legal consent was granted by the regional prosecutor.

### Quality control procedures

To minimize contamination, all solutions were filtered through 0.1 µm PTFE membrane filters (Merck Millipore, Burlington, USA). Personnel wore cotton gowns, surgical masks, and gloves. Samples were collected using ceramic instruments, rinsed with ultrapure water (resistivity 18.2 MΩ·cm; Millipore Direct-Q 3UV-R, Merck, Darmstadt, Germany), and stored in acid-washed glass Petri dishes. Samples were frozen at −80 °C until further analysis. Procedural blanks and control samples were processed in parallel and analyzed using SEM–EDS to ensure sample integrity.

### Sample digestion and filtration

Tissues were digested in 100 mL of 30% H₂O₂ at 50 °C under shaking conditions (80 rpm) for approximately two weeks (Jenner et al. 2022; Munno et al. 2018). The digested material was vacuum-filtered using aluminum oxide membrane filters (0.02 µm, Anodisc, Watford, UK). Filters were stored in glass Petri dishes for further microscopy, while the filtrates were lyophilized prior to spectrometric analysis. 

### Optical microscopy

Microscopic evaluation was performed using a VHX-7000 4K digital microscope (Keyence, Mechelen, Belgium), with magnification up to 6000×. Samples were observed in perpendicular, mixed, and transmitted light on aluminum oxide filters.

### SEM–EDS analysis

Morphological and elemental analyses were carried out using a FIB-SEM Scios 2 LoVac microscope (Thermo Fisher Scientific) equipped with an UltraDry Premium EDS system. Samples were mounted on aluminum stubs using carbon conductive tape and analyzed in low vacuum mode without sputtering. Imaging parameters were set at 15 kV and 0.8 nA using a segmented concentric backscattered electron detector (CBS) to differentiate composite and topographic contrasts.

### Optical photothermal infrared (O-PTIR) microscopy

O-PTIR spectra were obtained using the mIRage™-LS infrared and Raman microscope (Photothermal Spectroscopy Corp., Santa Barbara, CA, USA) equipped with a pulsed QCL (1844–940 and 3000–2698 cm⁻^1^) and a 532 nm CW probe laser. Data were collected in reflection mode using a 40× Cassegrain objective (NA 0.78). The QCL duty cycle was set to 1% (100 kHz), with laser power <45% and probe power <6.7% to avoid sample degradation. Spectral resolution was 6.6 cm⁻^1^ and scan rate 200 cm⁻^1^/s. Data were processed using PTIR Studio software v4.6.

### Dynamic light scattering (DLS)

Particle size distribution in filtrates was measured at 23 °C using a Zetasizer Nano ZS (Malvern Panalytical, Malvern, UK).

### MALDI-TOF mass spectrometry

Lyophilized filtrates (~20 mg) were sealed in glass ampoules and thermally degraded at 300 °C for 10 min (Lin et al. 2020). After cooling, the residues were dissolved in tetrahydrofuran (THF, 40 mg/mL; Sigma-Aldrich, USA). Matrix solution (1,8,9-anthracenetriol, 20 mg/mL) and Ag-TFA (10 mg/mL) were prepared fresh in THF and mixed in a 1:10:1 (sample:matrix:Ag-TFA) ratio. Aliquots (1 µL) were deposited onto a stainless-steel MALDI target (Waters Corp., Milford, MA, USA) in quadruplicate.

MALDI-TOF MS analysis was performed using a SYNAPT G2-Si HDMS system (Waters Corp.) equipped with a 1 kHz Nd:YAG laser. Spectra were acquired in positive ion mode (50–5000 Da), with external calibration using red phosphorus. Acquisition parameters included 350 V laser energy, 1000 Hz laser frequency, and 1 scan/s for 120 s.

## Results

### Screening by optical microscopy

**Fig. 1:**
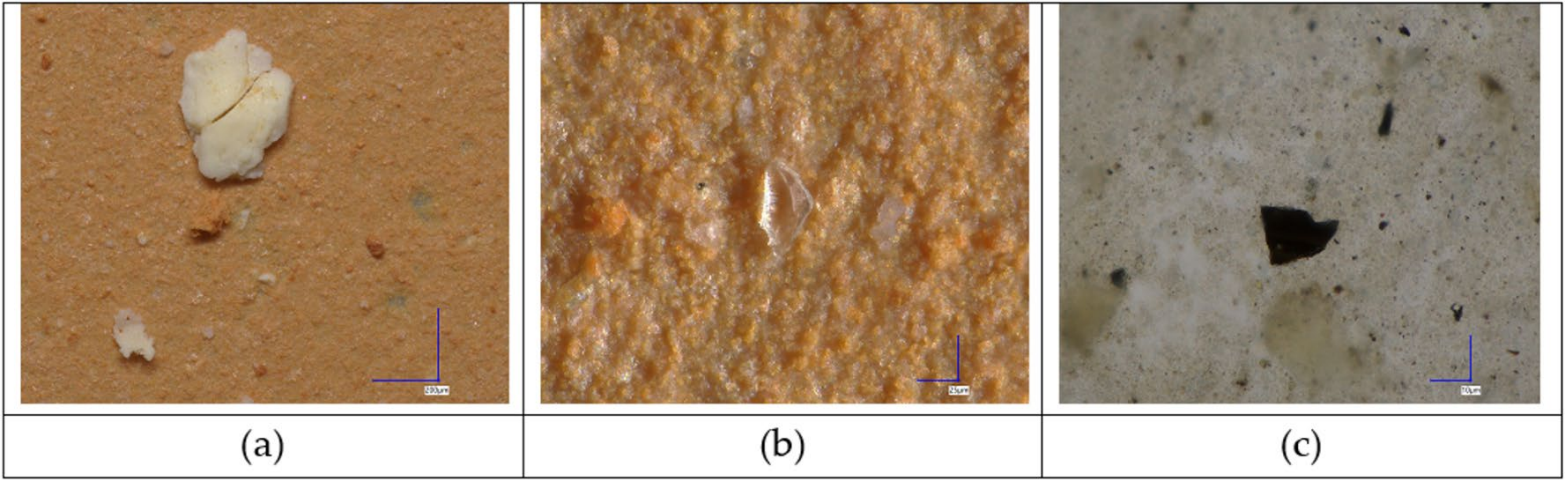
Multigranular aggregate of mineral substances in liver (**a,b**) and lungs (**c**) tissues seen under an optical microscope

After enzymatic digestion and vacuum filtration, various microparticles—including mineral substances, metals, and polymer-like materials—were retained on alumina filters. Mineral components appeared as single crystals or multi-granular aggregates, typically white in color. Orange–brown agglomerates of varying sizes suggested the presence of iron oxides (Fig. 1).

**Fig. 2:**
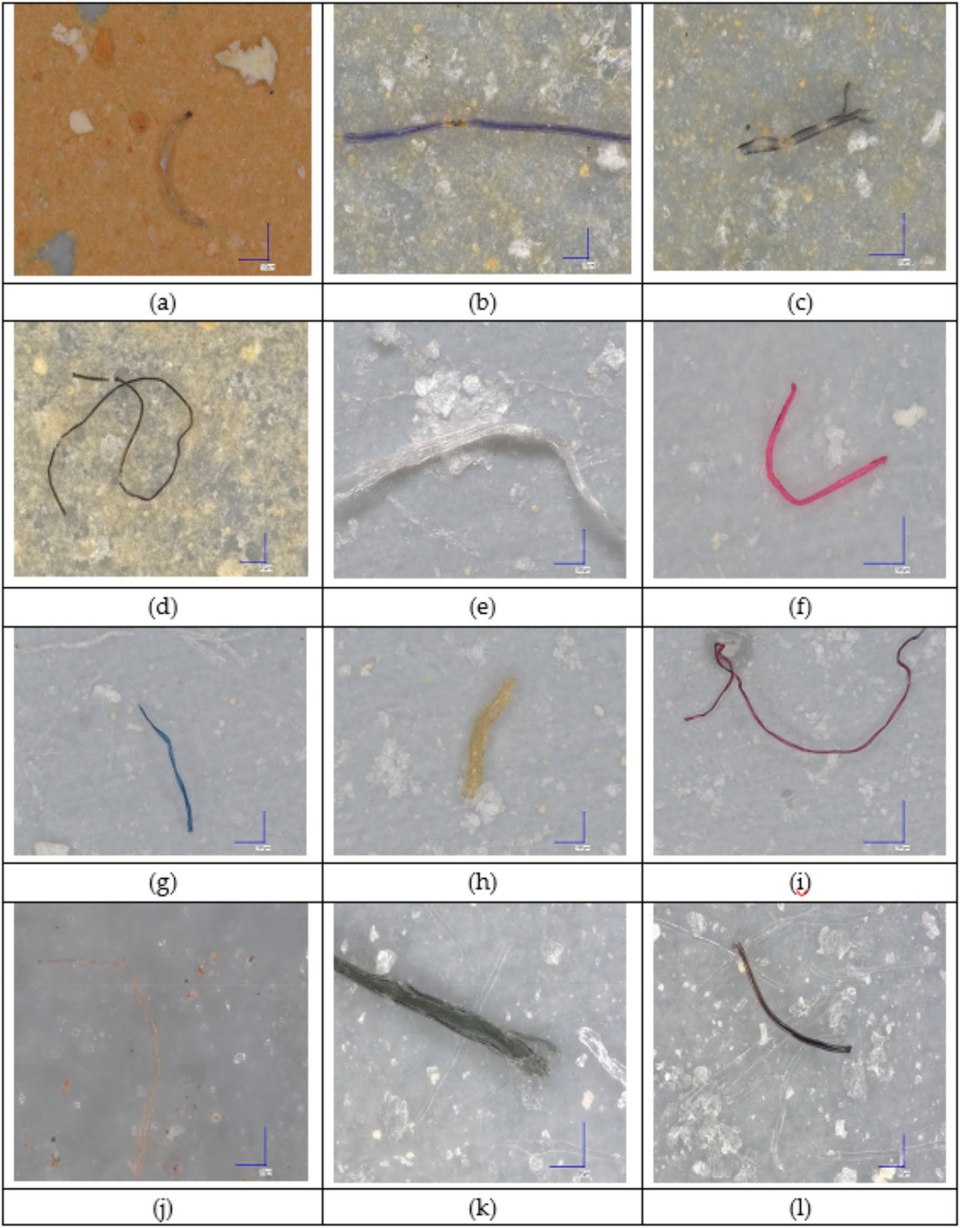
Optical microscopy images of plastic fiber extracted from tested tissues of the lung, liver, kidney, heart, thyroid, brain stem

Microplastic-like particles exhibited considerable variation in shape (fibrous, irregular, spherical), size, and color (white, transparent, yellow, red), indicative of different sources and degrees of environmental degradation (Fig. 2).

### Particle analysis using SEM/EDS

**Fig. 3:**
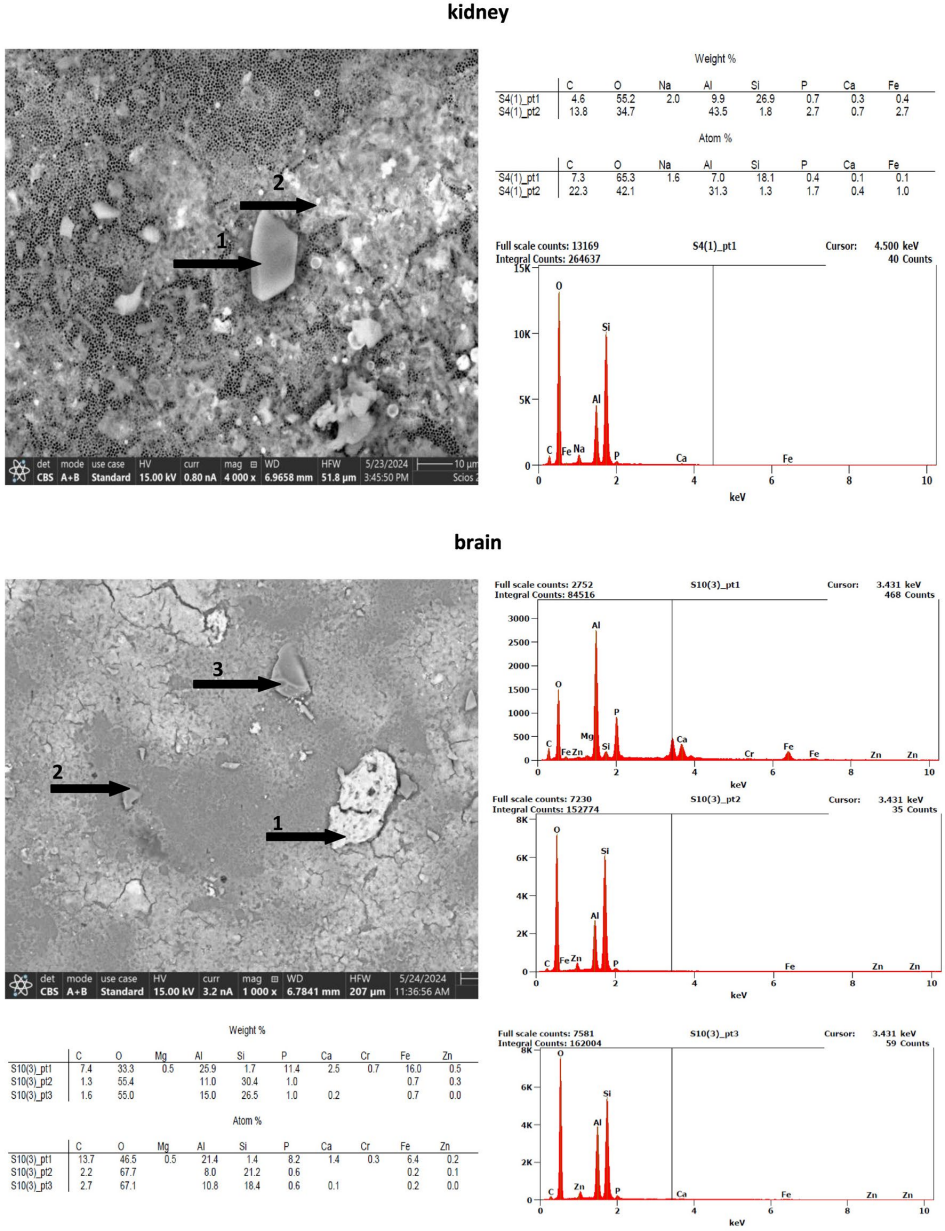
SEM/EDS images and spectra of selected particles isolated from human tissue samples (kidney and brain)

**Fig. 4:**
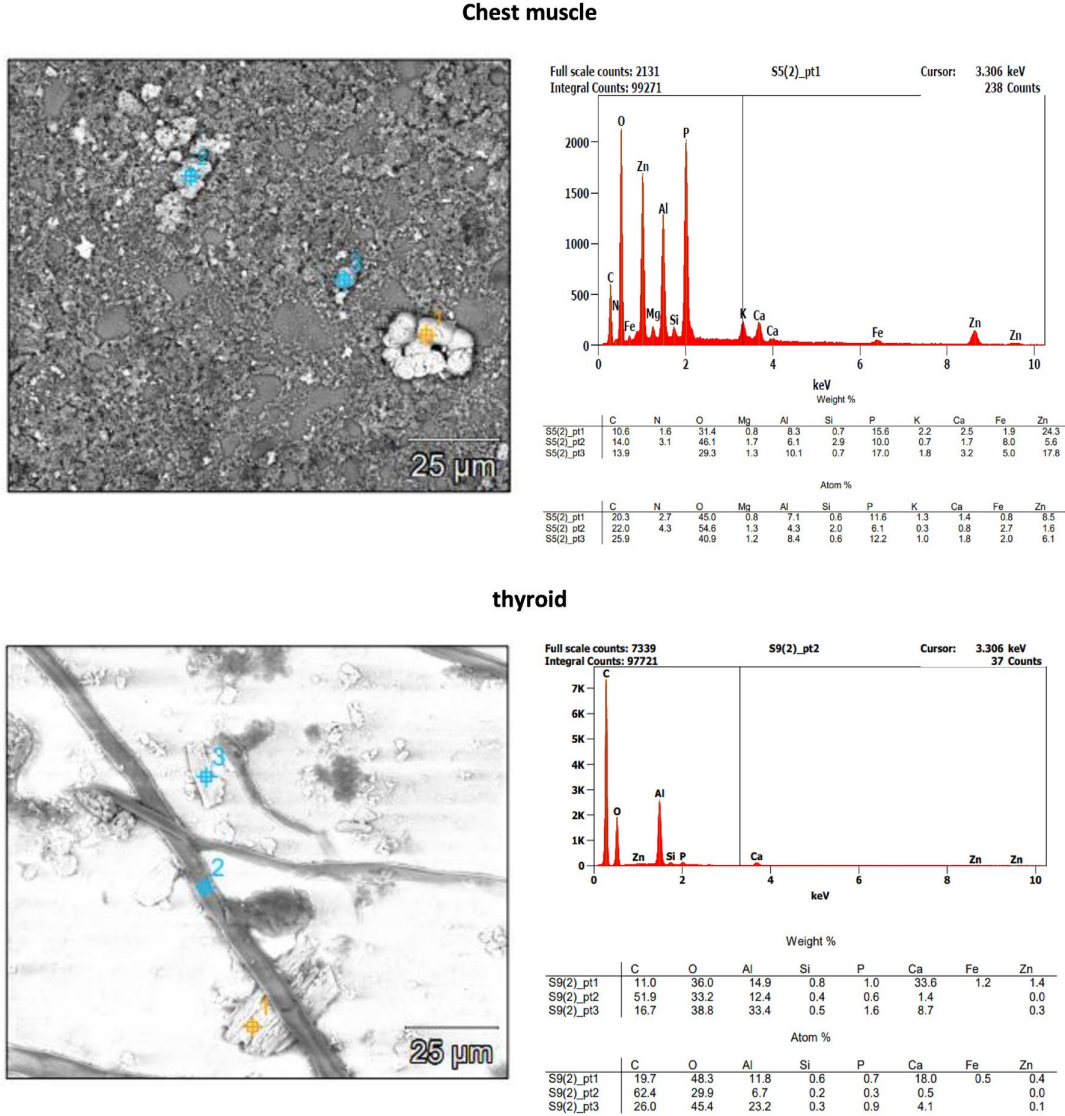
SEM/EDS images and spectra of selected particles isolated from human tissue samples (chest muscle and thyroid)

Optical microscopy was insufficient to confirm the composition of the observed particles. Therefore, elemental analysis using SEM–EDS **(**Figs. 3 and 4**)** was conducted. Inorganic particles displayed strong peaks for calcium (Ca), phosphorus (P), oxygen (O), silicon (Si), and aluminum (Al), consistent with carbonates, silicates, and aluminosilicates. Iron oxides and hydroxides were also detected. Carbon-rich particles lacking nitrogen (N) were observed as elongated, irregular structures with abraded surfaces—morphological indicators of polymeric origin. These exhibited a dominant carbon (C) signal and minor traces of Zn, Si, and P. Such features are consistent with environmentally aged microplastics (Zbyszewski et al. 2014). Examples of tissue-specific EDS profiles include: Thyroid: Dominant C peak, elongated shape, surface scratches; Kidney: Peak indicating silicon-rich particles; Brain stem: Complex mineral content with P, Si, Al, Zn, Fe, Mg; Pectoralis muscle: Abundant microelements (Zn, Al, P, Ca, K, Fe).

### Polymer identification by O-PTIR microscopy

O-PTIR analysis confirmed the presence of synthetic and semi-synthetic polymers among the microparticles isolated from all examined tissues (Table 1). The method enabled precise, non-destructive chemical identification of submicron particles directly on the filter surface.

**Table 1:** Polymers identified in human tissues using O-PTIR microscopy

Tissue	The mass of sample (g)	The number of MPs	The kind of MPs	The shape of MPs	The range of lengths, widths (μm)	MPs per g of tissue
Liver	2.12	14	CE, VT, PET	Fibers	206–740, 9–26	6.6
Lung	1.59	18	CE, VT, PET, RE	Fibers	428–2060, 10–23	11.3
Kidney	0.93	20	PS, CE, VT, PC	Fibers	250–1504, 17–30	21.5
Chest muscle	2.11	11	PAN, CE, PVC, PET, PI	Fibers	485–1740, 8–27	5,2
Heart	1.03	13	CE, NB, PVC, PET	Fibers	220–3796, 6–25	12,6
Thyroid	1.09	44	CE, VT, PC, PS, NB, PET, PI, PVC, PLA,	Fibers	228–3265, 11–28	40.4
Brain	0.82	20	PET, CE, PI, PLA	Fibers	230–1758, 8–21	24.4

**Fig. 5:**
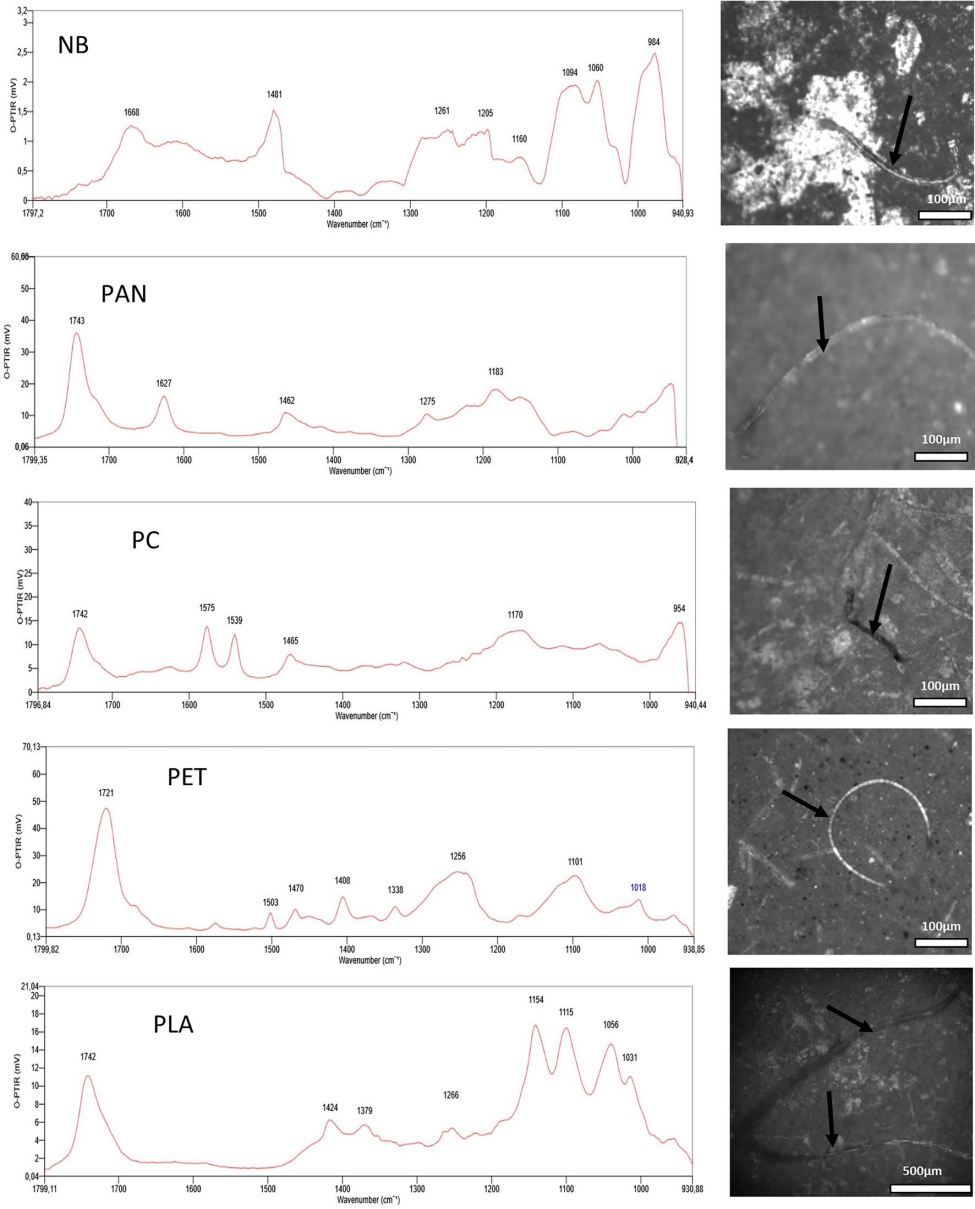
Example of MPs (NB, PAN, PC, PET, PLA) identified within tissue samples by O-PTIR microscope

**Fig. 6:**
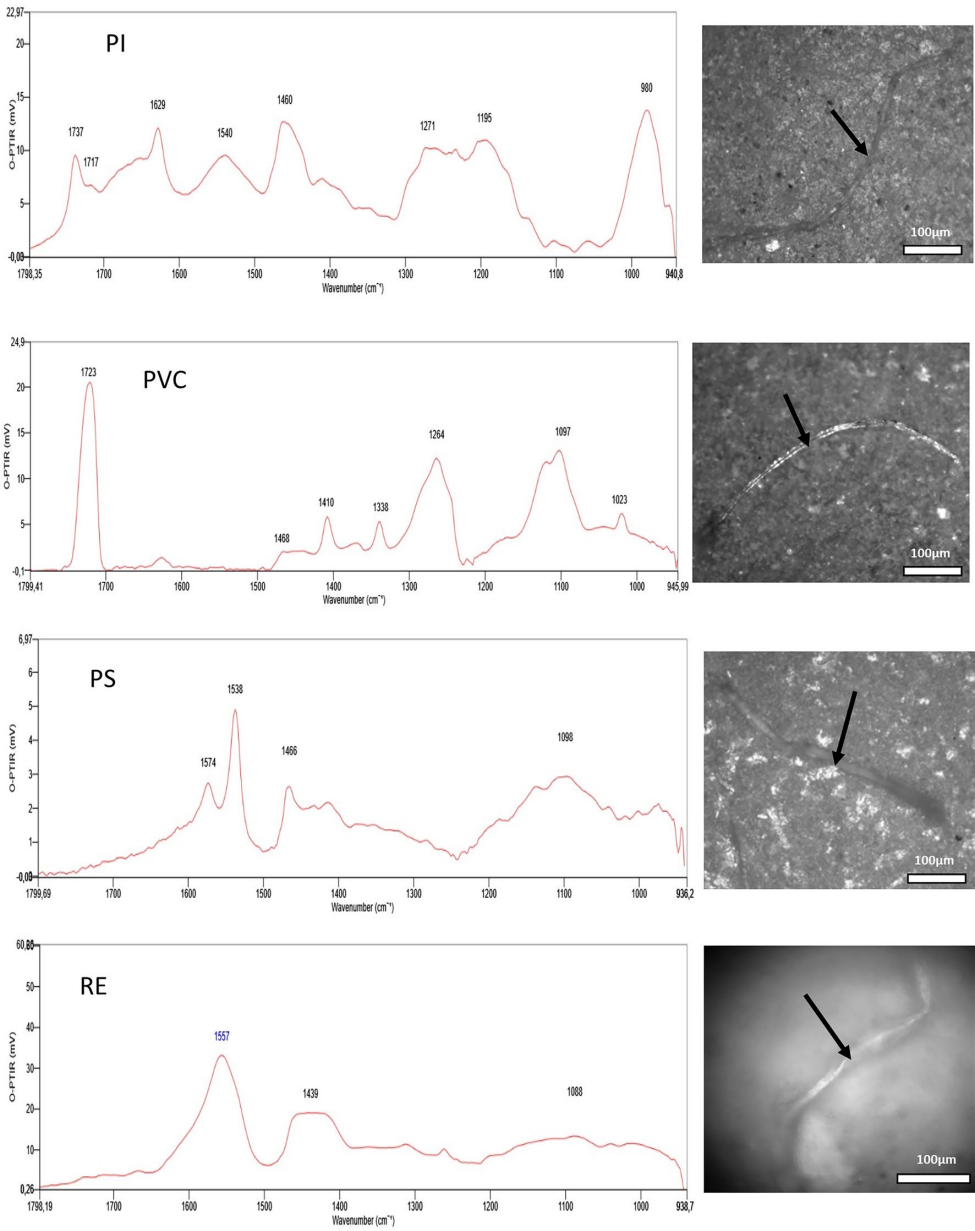
Example of MPs (PI, PVC, PS, RE) identified within tissues samples by O-PTIR microscope

**Fig. 7:**
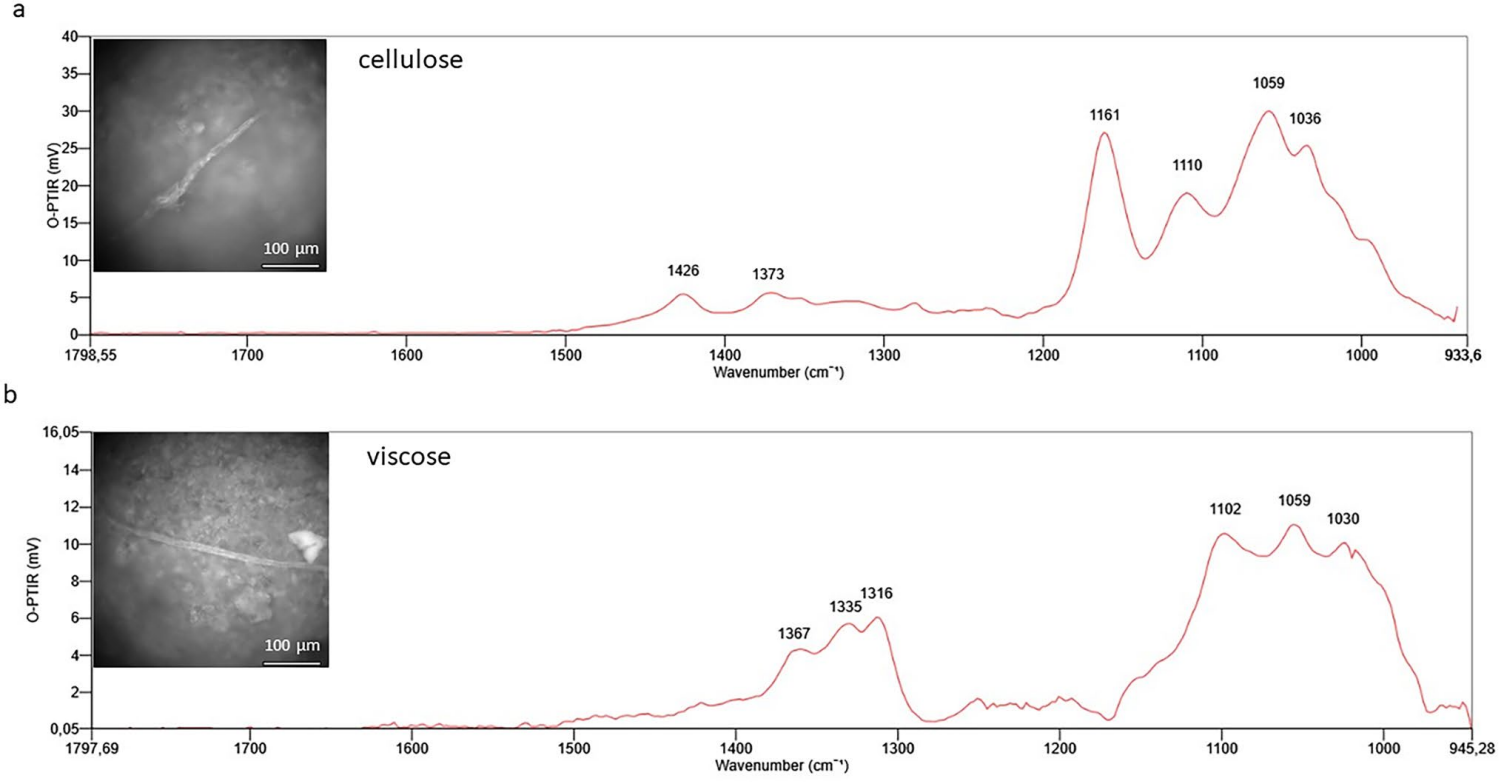
O-PTIR spectra of cellulose and viscose type microparticles

Representative O-PTIR spectra for key polymer classes are presented in Figs. 5, 6 and 7. The identified synthetic polymers include:

1. NB (nylon blend): Spectra of nylon blends were identified by characteristic bands of amide I (C=O stretching bands) and amide II (N–H bending bands) vibrations in the range of 1670–1500 cm^−1^. In addition, the spectra confirm the presence of vibrations characteristic of CH2 group at 1480 cm^−1^ and CH as well as C–C in the range of 1300–1000 cm^−1^ (Tarafdar et al. 2024).
2. PAN (polyacrylic resin/polyacrylonitrile): In the case of PAN, all absorption bands characteristic of polyacrylonitrile were clearly visible at 1743 cm^−1^ (C=O stretching), 1627 cm^−1^ (C=C stretching), 1463 cm^−1^ (CH bending in CH2) and 1275 cm^−1^ (Rajzer et al. 2012).
3. PC (polycarbonate): The spectra of PC typically display the presence of a C=O stretching vibration band at around 1742 cm^−1^. Moreover, a characteristic feature of IR spectra for PC is also the presence of vibrational bands in the wavenumber range 1600–1500 cm^−1^ attributed to vibrations of the aromatic ring. The band at 1465 cm^−1^ indicates bending vibrations of CH2 groups. Whereas, bands around 1200–1100 cm^−1^ originate from the stretching vibrations of the C–O ester groups (Böke et al. 2022).
4. PET (polyethylene terephthalate): The IR PET spectra display characteristics band at 1721 cm^−1^, which is responsible for C=O stretching. The observed weak bands between 1650–1450 cm^−1^ indicate vibrations of the ring and CH groups. The band at 1408 cm^−1^ reflects C–C phenyl ring stretching. The band at 1256 cm^−1^ and the band at 1102 cm^−1^ display stretching of the aromatic C–C-O/O–C–C ester. Moreover, the characteristic band at 1018 cm^−1^ also occurs and represents CH bending (Liang and Krimm 1959; Böke et al. 2022; Lin et al. 2024).
5. PLA (polylactic acid): The vibrations characteristic of the C–O ester bond and their coupling effects are revealed in the range of 1200–1000 cm^−1^, while the bands located at 1424 and 1379 cm^−1^ originate from the deformation vibrations of CH2 and CH3 groups. In addition, the stretching vibration of C=O groups is revealed at 1742 cm^−1^ (Böke et al. 2022).
6. PI (polyimide): Characteristic bands for PI include a stretching vibration of C=O group, two characteristic bands (one from carboxylic acids and one from ketones) in the range of 1750–1700 cm^−1^. In addition, vibration from the aromatic ring in the range of 1650–1400 cm^−1^, and a band from the C-N stretching vibration at 1270 cm^−1^ are noticeable (Snyder et al. 1989).
7. PVC (poly(vinyl chloride)): The vibrations observed at 1097 and 1264 cm^−1^ originate from stretching vibrations of the C–C and C–O bonds. The peak at 1264 cm^−1^ is also attributed to wobble vibrations of the neighboring CH2 carbon atom, which is bonded to a chlorine atom. The bands observed at 1400–1470 cm^−1^ are characteristic of deformation vibrations of CH2 groups. The peak at 1723 cm^−1^ is derived from C=O stretching vibrations of plasticizers in PVC (Böke et al. 2022; Tarafdar et al. 2024).
8. PS (polystyrene): The identified vibration bands that we can attribute to PS are responsible for the vibration of the aromatic ring at 1600–1500 cm^−1^ and characteristic “benzene fingers” from 1650 to 1800 cm^−1^ are present. The next band characteristic of PS is the one at 1466 cm^−1^, which is responsible for methylene vibrations (C–H bending) (Böke et al. 2022; Tarafdar et al. 2024).
9. RE (resin alkyd/epoxy/hydrocarbon): another group of polymeric compounds present in the samples are resins with characteristic bands in the range of 1600–1500 cm^−1^ indicating stretching vibration of the aromatic ring, and stretching vibration for C–C at 1440 cm^−1^. In addition, the presence of a broad vibration band at 1080 cm^−1^ indicates stretching vibration of C–O–C (Tarafdar et al. 2024).

According to (Baeza-Martínez et al. 2022), the vast majority of MPs detected in the lower airways were semisynthetic cellulose derivatives (40.48%) and cellulose plus cotton (31%). This was confirmed in our study, where cellulose and/or its semi-synthetic derivatives (viscose) were found in all 7 types of tissues analyzed. O-PTIR spectra and microphotographs for CE and VT are shown in Fig. 7.

1. CE (cellulose fibers): The identified O-PTIR bands at 1426 cm^−1^ can be ascribed to the CH2 bending of pyranose ring, while band at 1371 cm^−1^ can be assigned to CH deformation. Moreover, bands at 1161, 1059 and 1036 we can attribute to C–O stretching vibrations, and band at 1110 cm^−1^ represents ring asymmetric stretching (Carrillo et al. 2004; Comnea-Stancu et al. 2017; Baeza-Martínez et al. 2022).
2. VT (viscose type fibers): The biggest difference between natural cellulose fibers and fibers of viscose type is the absence of a band or the presence of a weak intensity band or shoulder at 1160 and 1420 cm^−1^. Other characteristic bands for polysaccharides at roughly 1380–1320 (CH deformation) cm^−1^ and 1100–1000 cm^−1^ (C–O stretching vibrations) are present (Baeza-Martínez et al. 2022).

### DLS analysis of tissue filtrates

Dynamic light scattering (DLS) analysis confirmed the presence of polydisperse nanoparticle populations in all tissue filtrates (Table 2). The polydispersity index (PDI) ranged from 0.374 to 0.923, and hydrodynamic diameters ranged from 340 to nearly 2000 nm, indicating aggregation tendencies. Brain, thyroid, and pectoral muscle samples exhibited the most complex nanoparticle profiles.

**Table 2:** Particle size distribution in tissue filtrates obtained via DLS

Sample	Z-Ave d.nm	PdI	Pk 1 Mean Int. d.nm ± st.dev	Pk 2 Mean Int. d.nm ± st.dev	Pk 3 Mean Int. d.nm ± st.dev	Pk 1 Area Int [%]	Pk 2 Area Int [%]	Pk 3 Area Int [%]
Liver	712.5	0.645	245.8 ± 30.5	0	0	100	0	0
Muscle	642.7	0.719	480.3 ± 176.8	106.2 ± 35.6	1853 ± 6.1e^−5^	77.9	20.7	1.3
Heart	2396	0.923	250.2 ± 23.3	0	0	100	0	0
Thyroid	382.5	0.459	334.9 ± 70.5	328.9 ± 72.6	0	66.0	33.9	0
Brain	1999.7	0.889	129.8 ± 29.2	12.5 ± 3.8	0	97.1	2.9	0
Lungs	721.6	0.589	155.0 ± 22.7	0	0	100	0	0
Kidney	341.6	0.374	148.0 ± 30.1	0	0	100	0	0

### MALDI-TOF mass spectrometry of filtrates

**Fig. 8:**
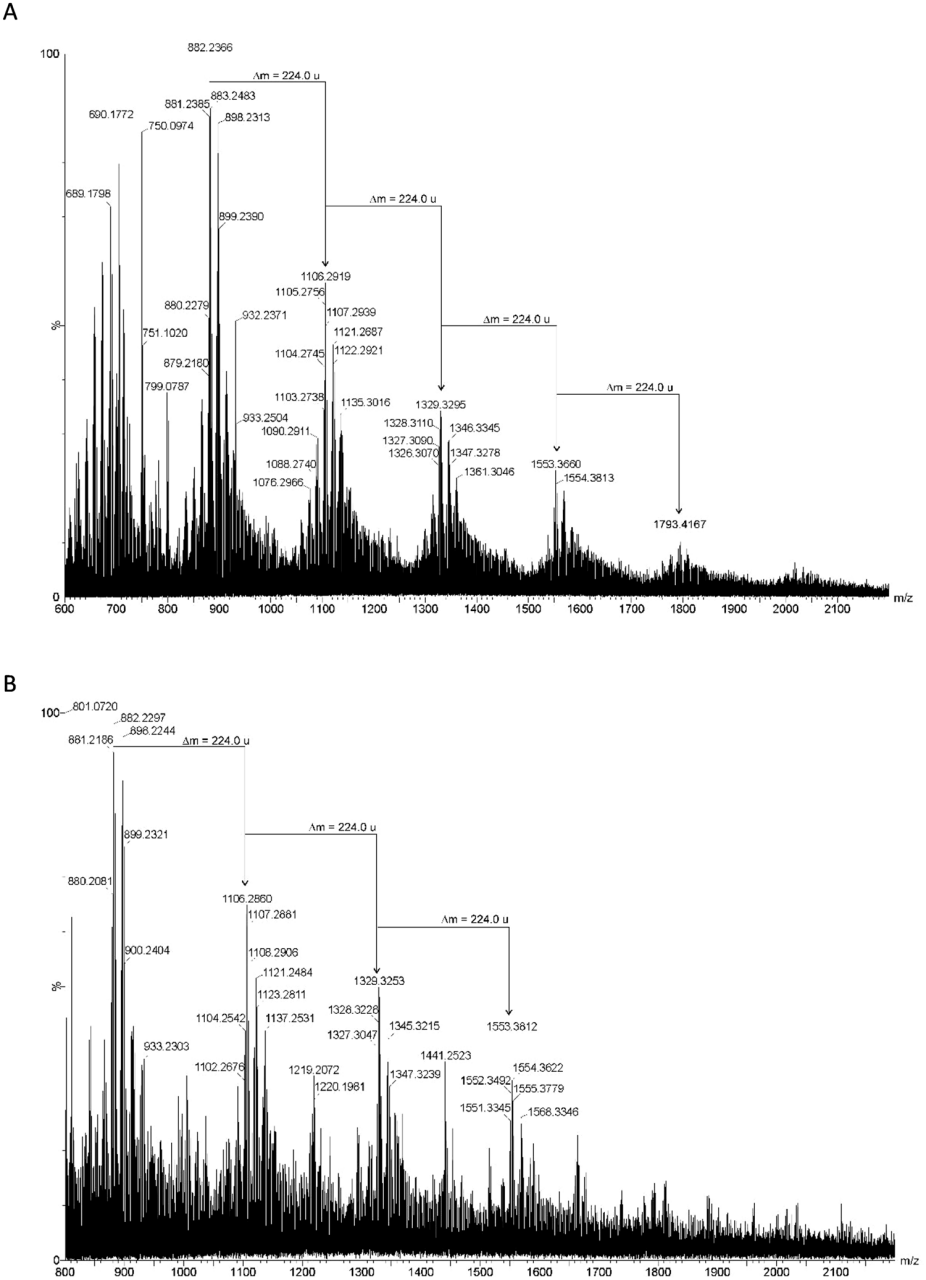
Mass spectrum (MALDI-TOF MS, positive ion mode) of filtered obtained from the lung tissue (**A**) and white matter of the brain (**B**). The mass differences visible at the spectrum (Δm = 224.0 u) derived from oxygenated PS Ag-derivatives

**Fig. 9:**
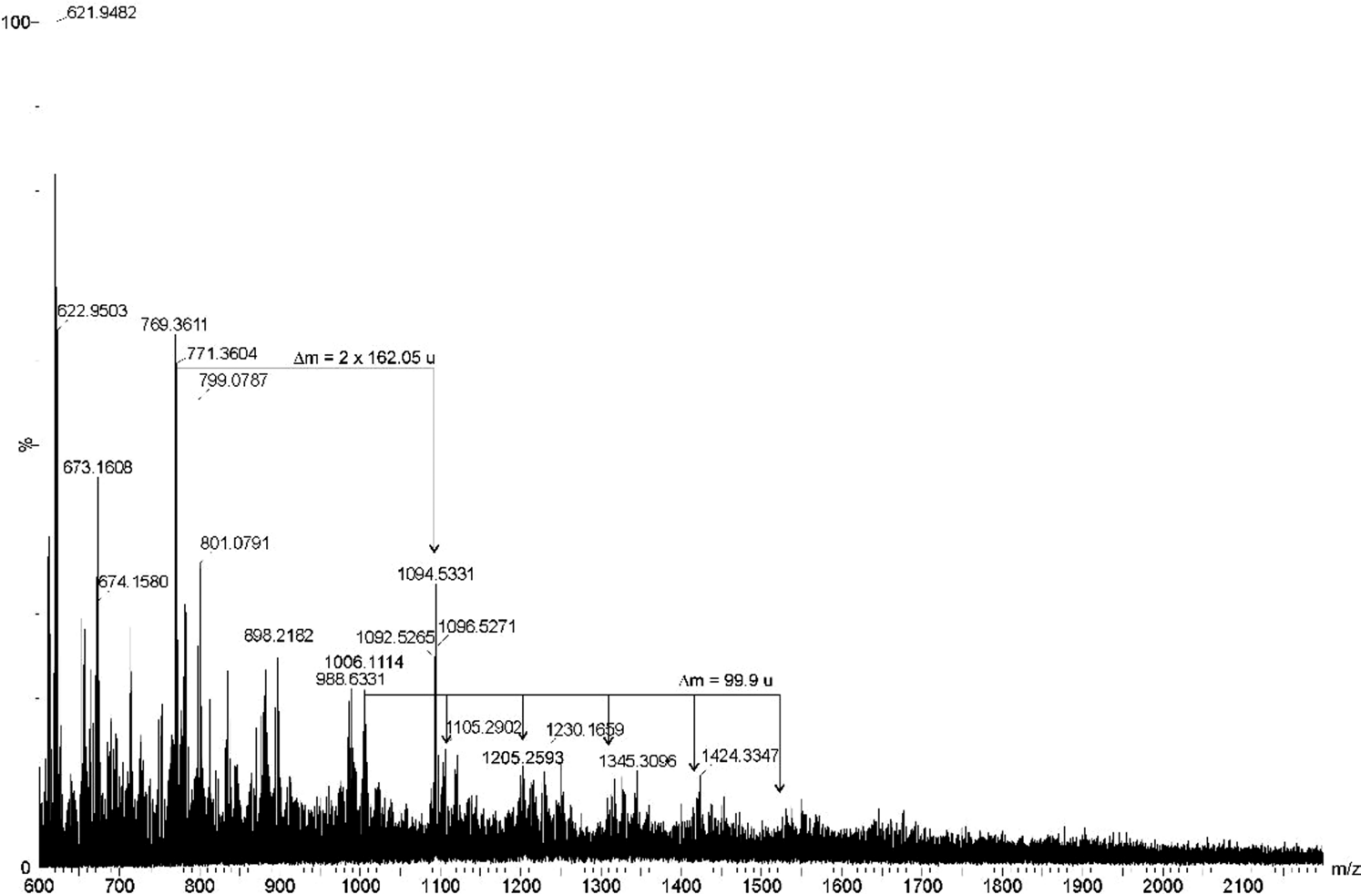
Mass spectrum (MALDI-TOF MS, positive ion mode) of filtered obtained from the kidney tissue. The mass differences of about 99.9 u derived from PET, and fragments with mass differences at 162.05 indicated the presence of CE

**Fig. 10:**
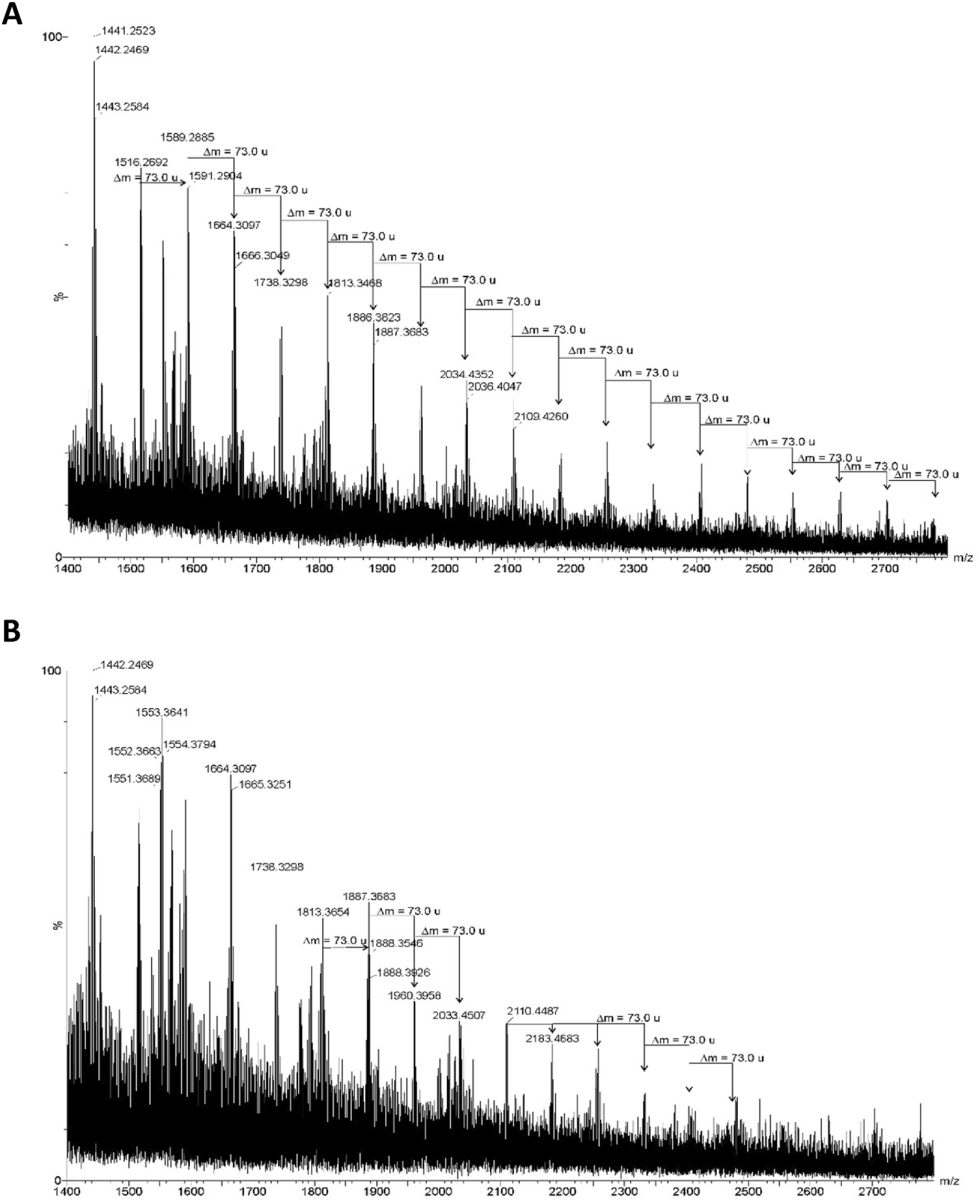
Mass spectrum (MALDI-TOF MS, positive ion mode) of filtered obtained from the thyroid tissue (**A**) and heart tissue (**B**). The mass differences visible at the spectra (Δm = 73.0 u) derived from fragments of PAN resin present in these samples

Thermally pre-treated filtrates were analyzed by MALDI-TOF MS to confirm polymeric composition. Figures 8, 9, 10 illustrate MALDI-TOF MS spectra of selected filtrates and interpretation of polymer signatures.

In brain and lung samples, mass spectra revealed peaks in the 600–2000 m/z range with a characteristic mass increment of 224.0 u, corresponding to [C₁₆H₁₆O] repeat units and indicative of polystyrene-silver (PS–Ag) derivatives (Lin et al. 2020; Wu et al. 2020) (Fig. 8).

In kidney filtrates, a cluster spacing of ~99.9 u confirmed the presence of PET oligomers. Additionally, mass differences of 2 × 162.05 u suggested cellulose-derived hexose fragments (Fig. 9).

Spectra from heart and thyroid filtrates showed broad series with ~73 u spacing, consistent with PAN resin oligomers (Fig. 10).

## Discussion

This study demonstrates the presence of micro- and nanoplastics (MNPs) in various post-mortem human tissues. The heterogeneity in particle type, size, and abundance among organs indicates that MNP translocation and retention are tissue-specific processes.

### Bioaccumulation across tissues

All examined organs contained detectable levels of synthetic and semi-synthetic microparticles, with the highest accumulation in the thyroid (40.4 MP/g), followed by the brain and kidneys. This pattern suggests differential tissue affinity and physiological barriers affecting MNP distribution. Our results are consistent with previous studies indicating organ-specific bioaccumulation in the lungs, colon, placenta, and bloodstream (Ragusa et al. 2021; Jenner et al. 2022; Horvatits et al. 2022; Zhu et al. 2023; Rotchell et al. 2023).

Notably, this study is the first to report the presence of MNPs in human thyroid tissue. While prior animal studies have shown endocrine disruption following MNP exposure (Zhang et al. 2024), the current data provide direct evidence of potential accumulation in human endocrine organs.

### Sample origin and methodological considerations

Unlike most studies based on samples from surgical procedures—which may introduce plastic contamination (Yang et al. 2023)—this study was conducted using autopsy material under strictly controlled conditions. The slow digestion protocol, based on gradual H₂O₂ oxidation at 40–65 °C, minimized potential polymer degradation, particularly of PS, PAN, and PE fibers. Additionally, analysis of the filtrates allowed for the detection of nanoplastics (PS, PET, PAN, CE) as small as 20 nm, providing novel insight into the nanoscale fraction of contamination.

Limitations include the small sample size and potential environmental contamination despite strict quality control. Nonetheless, the inclusion of seven tissues from a single donor allows for a reliable comparison of organ-specific accumulation.

### Polymer types and nanoplastic fraction

O-PTIR and MALDI-TOF MS identified various synthetic polymers including PS, PET, PAN, PVC, and PLA, as well as natural and semi-synthetic fibers such as cellulose (CE) and viscose (VT). The detection of PS nanoplastics in the filtrates of brain, lungs, kidney, heart, and thyroid tissues is particularly concerning, given their small size (<20 nm), which increases systemic bioavailability, cellular uptake, and potential toxicity (Tomazic-Jezic et al. 2001; Buzea et al. 2007).

CE and nanocellulose particles were also detected, especially in kidney samples. While nanocellulose is considered biocompatible and is widely used in biomedical and food applications (Elfaleh et al. 2023; Malekpour et al. 2023), its long-term health effects— especially at the nano scale—remain insufficiently studied. Some studies have suggested oxidative and inflammatory responses (Brand et al. 2022), though evidence of genotoxicity is still lacking.

### Functional implications

The presence of MNPs in the brain and heart supports the hypothesis of translocation via the circulatory system. Previous studies detected PET, PS, and PE in human blood and vascular tissues (Leslie et al. 2022; Rotchell et al. 2023). The observation of PS, PET, and CE nanoparticles in brain tissue suggests potential penetration across the blood–brain barrier. In mouse models, such particles have been associated with neurotoxicity, endocrine disruption, and inflammation (Chen et al. 2021; Zhang et al. 2024).

In kidneys, 21.5 MP/g were detected, consistent with prior reports of MPs in urine and experimental models confirming renal accumulation and damage (Deng et al. 2017; Meng et al. 2022; Pironti et al. 2023). Similarly, MPs in lung samples measured in our study (fibers 10–23 µm wide, 428–2060 µm long) align with previous findings, although shorter particles have been more frequently reported (Jenner et al. 2022).

Skeletal muscle showed the lowest MNP content (5.2 MP/g), but existing animal studies suggest potential impacts on muscle regeneration, oxidative stress, and inflammation (Shengchen et al. 2021). To date, no human studies have investigated microplastic effects on the muscular system.

### Broader implications and future directions

The detection of diverse MNP types—including hazardous substances like PS and PVC—and their presence in all major organ systems underscores the need to reassess human exposure pathways and toxicological impacts. While most studies focus on feces, blood, or urine due to non-invasive access, tissue-level monitoring—particularly post-mortem—provides unique insights into internal accumulation and bioavailability.

This study reinforces the importance of developing standardized protocols for tissue digestion and particle detection, and highlights the need for further research into the long-term health consequences of chronic MNP exposure in humans.

## Conclusions

In our study, we applied a combination of O-PTIR imaging, SEM–EDS, and optical microscopy to identify and characterize microparticles in post-mortem human tissues. The results show that the ability of different organs to accumulate synthetic and natural polymer particles varies significantly. The thyroid, kidney, and brain exhibited the highest levels of microplastic accumulation, suggesting a heterogeneous pattern of translocation and retention within the human body.

Microparticles detected on the filters included fibers with widths below 30 µm, while the filtrates contained nanoparticles ranging from 300 to 2000 nm. The analysis of these filtrates—performed here for the first time—provided additional insight into the nanoscale fraction of plastic contamination.

Our findings underscore the need for further research to understand the mechanisms of micro- and nanoplastic uptake, tissue affinity, and systemic distribution. This knowledge will be essential for developing future strategies to reduce human exposure and prevent accumulation of plastic particles in internal organs. 

## Data Availability

The datasets generated during and/or analysed during the current study are available from the corresponding author on reasonable request.
